# Survival in Elderly Ovarian Cancer Remains Challenging in the Nordic Countries

**DOI:** 10.3390/cancers16122198

**Published:** 2024-06-11

**Authors:** Kari Hemminki, Frantisek Zitricky, Asta Försti, Akseli Hemminki

**Affiliations:** 1 Biomedical Center, Faculty of Medicine in Pilsen, Charles University in Prague, 30605 Pilsen, Czech Republic; frantisek.zitricky@lfp.cuni.cz; 2 Division of Cancer Epidemiology, German Cancer Research Center (DKFZ), Im Neuenheimer Feld 580, D-69120 Heidelberg, Germany; 3 Hopp Children’s Cancer Center (KiTZ), 69120 Heidelberg, Germany; a.foersti@dkfz.de; 4 Division of Pediatric Neurooncology, German Cancer Research Center (DKFZ), German Cancer Consortium (DKTK), 69120 Heidelberg, Germany; 5 Cancer Gene Therapy Group, Translational Immunology Research Program, University of Helsinki, 00290 Helsinki, Finland; akseli.hemminki@helsinki.fi; 6 Comprehensive Cancer Center, Helsinki University Hospital, 00290 Helsinki, Finland

**Keywords:** prognosis, relative survival, treatment, carboplatin cancer control

## Abstract

**Simple Summary:**

Ovarian cancer has been the most fatal female-specific cancer, and in particular, old patients have worse survival compared to young patients. In this study, we test whether the current oncology practice has been able to improve survival, compared to age group-specific past survival. We were able to show that survival in old patients remains poor, and in all patients, the time after year 1 is a critical period for survival. Although survival in ovarian cancer has improved in the Nordic countries over the past 50 years, age-related disparities have remained, or even increased. Survival has not increased for patients older than 60 years after one year of survival. This study calls for more focus on elderly ovarian cancer patients, to aim for early diagnosis and to explore more active therapeutic intervention for which age should not be considered limiting but rather the physical condition.

**Abstract:**

Background: Despite treatment having improved through intensive surgical procedures and chemotherapy—and more recently, targeted therapies—ovarian cancer is the most fatal female cancer. As such, we wanted to analyze age-specific survival trends for ovarian cancer in Denmark, Finland, Norway and Sweden over the past 50 years, with a special aim of comparing survival development between the age groups. Methods: We modelled survival data from the NORDCAN database for 1-, 5- and conditional 5/1-year relative (between years 1 and 5) survival for ovarian cancer from 1972 to 2021. Results: Young patients had a 70% 5-year survival while the survival was only 30% for the oldest patients. Conditional survival showed that survival between years 1 and 5 did not improve for patients older than 60 years throughout the 50-year period, during which time the gaps between the youngest and the oldest patients widened. Conclusions: Improvement in 1-year survival was so large that it masked the modest development between years 1 and 5, resulting in a widening age disparity in 5-year survival. The current treatment practices, which appear increasingly effective for younger patients, have not helped remedy the large age differences in ovarian cancer survival. Early detection methods and therapeutic innovations are urgently needed, and aged patients need a special focus.

## 1. Introduction

Epithelial ovarian cancer (also called tubo-ovarian carcinoma, here ‘ovarian cancer’) accounts for more than 90% of ovarian malignancies [1]. It ranks as the third most common female-specific cancer after breast and endometrial cancers in Northern Europe [2]. Important risk factors for ovarian cancer include obesity, infertility, nulliparity and estrogen hormone treatment, whereas high parity, breastfeeding and oral contraceptive use are protective factors [3]. Germline mutations in BRCA1/2 and mismatch repair genes predispose to ovarian cancer and may in part contribute to the known familial risk [1,3,4]. BRCA1/2 mutations are associated with the most common histological type of high-grade serous carcinoma, while mismatch gene mutations often present as endometrioid and clear cell carcinomas [3]. FIGO staging is commonly used to describe tumor spread to the pelvic and peritoneal areas (stages II and III) or to distant organs (stage IV) [3]. Surgery has been the main treatment for ovarian cancer, with the aim to remove all suspicious lymph nodes in early stage disease and to conduct radical cytoreductive surgery for debulking (i.e., removal of as much tumor as possible) in the advanced disease [3,5]. Ovarian cancer has been one of the first tumor types where surgery is performed despite metastases; indeed, in ovarian cancer this has become a standard treatment [3].

In the Nordic countries, the 5-year relative survival of ovarian cancer was about 50% after 2015, which was the worst survival among the female cancers, in spite of the large improvement in past years [2,6]. Short-term survival improvements have been reported from the Netherlands, but no improvements in long-term survival could be confirmed [7]. In a global survival study, covering the years from 2010 to 2014, Sweden and Norway were in the top 5-year survival group for ovarian cancer (46.5% and 45.5%, respectively), only Costa Rica had a survival rate of over 50% [8]. Survival in the USA was 43.4%, 46.3% in Japan, and 47.5% in Korea [8]. However, a recent Korean publication on 5-year relative survival in ovarian cancer covering the overlapping years of 1999 to 2019 reported a survival of 62.2% [9].

In the present study, we assess the relative survival in ovarian cancer in the four Nordic countries of Denmark (DK), Finland (FI), Norway (NO) and Sweden (SE) over 50 years up to 2021, based on the NORDCAN database. We apply three relative survival metrics—1-, 5- and conditional 5/1-year survival—in order to focus on the changes in survival from the time of diagnosis; 5/1-year survival measures survival for those who survived the first year to survive additional four years. Health care in the Nordic countries has been organized according to the principle of population access with minimal direct out-of-pocket costs, thus the results describe a ‘real-world’ survival experience. NORDCAN has been set up by the Nordic cancer registries, which are the oldest national cancer registries in the world and are characterized by high quality and minimal loss to follow-up, which are important features for reliable survival estimation [10].

## 2. Methods

The data were obtained from NORDCAN database 2.0, assessed in the spring of 2024 [10,11] from the International Agency for Cancer (IARC) website (https://nordcan.iarc.fr/en, version 9.3, accessed on 12 April 2024) [12]. Data were extracted for a cohort study on incidence, mortality and 1- and 5-year survival. International Classification of Diseases (ICD) version 10 codes were used in NORDCAN to describe the tumor locations. The codes for ovarian cancer were C56 (malignant tumors of the ovary), and C57.0–C57.4 (tumors of the fallopian tubes, ligaments and adnexa).

The follow-up was terminated at death, emigration or loss of follow-up or by the end of 2021. The survival data were available from 1972 onwards; the analysis was based on the cohort survival method for the first nine 5-year periods, and the period approach was used for the last period 2017–2021 [13,14]. Relative survival was estimated using the Pohar Perme method [15]. Age standardization (Table 1 and Tables S2–S3) was performed by weighting individual observations using external weights as defined at the IARC website. Age groups 0 to 89 were considered. The national life tables were used to calculate the expected survival.

Statistical modelling and data visualizations were performed using R statistical software (https://www.r-project.org, accessed on 12 April 2024) in the R studio environment (https://posit.co/, version 2023.09.1, accessed on 12 April 2024). Trends in relative survival (1-year, 5-year and 5/1-year conditional survival) were modelled with Gaussian generalized additive models (GAM), as detailed [16]. The modelling was performed on a cumulative hazard scale, which allowed for the inclusion of asymmetric confidence intervals (CIs) provided by NORDCAN for each estimate. As the model input, survival estimates were assigned a timepoint in the middle of the respective period. The GAM models were run for each country and included the effect of age group and the non-linear effect of time, using thin plate regression splines (k = 4). The models were run in the Bayesian framework using the ‘brms’ R package [17,18], which employs ‘Stan’ software for probabilistic sampling [19].

Age-specific mortality was retrieved from the NORDCAN database. Note that mortality is cause specific, i.e., death in ovarian cancer. The age-dependent mortality rates were interpolated with smoothing splines (smooth.spline function from the ‘stats’ package in R, 6 knots).

Comparisons with the US Surveillance, Epidemiology and End Results (SEER) data for years 2013–2019 on White (including Hispanics) women were conducted through the following website: https://seer.cancer.gov/statistics-network/explorer/application.html?site=1&data_type=1&graph_type=2&compareBy=sex&chk_sex_3=3&chk_sex_2=2&rate_type=2&race=1&age_range=1&hdn_stage=101&advopt_precision=1&advopt_show_ci=on&hdn_view=0&advopt_display=2#graphArea, accessed on 12 April 2024.

Differences were assumed significant if their 95% CIs are non-overlapping.

## 3. Results

### 3.1. Case Numbers and Diagnostic Ages

The numbers of patients diagnosed with ovarian cancer in the first (1972–1976) and the last (2017–2021) 5-year period declined in DK, and particularly in SE, and increased in FI and NO (Table S1). The median age at onset increased by 6 years. Detailed incidence and mortality trends for all female cancers have been recently published [2].

### 3.2. Relative Survival

**Figure 1:**
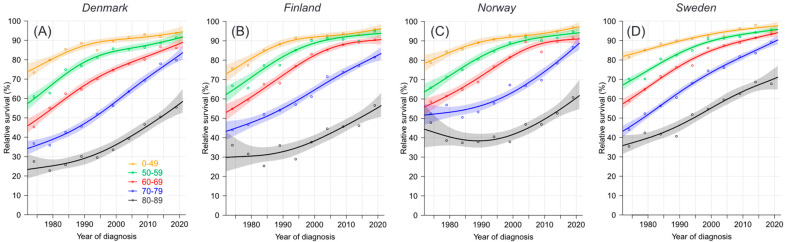
Relative 1-year survival in age groups with ovarian cancer in Denmark (**A**), Finland (**B**), Norway (**C**) and Sweden (**D**), 1972–1976 to 2017–2021. Data were modelled based on the NORDCAN database.

Relative age-specific 1-year survival in ovarian cancer in the Nordic countries over the past 50 years is shown in Figure 1. The starting levels for all age groups were lower in DK and FI than those in NO and SE, and small differences between these countries remained to the end, particularly in the oldest age groups. SE survival in the oldest age groups reached the highest levels, close to 70% for the 80–89-year-olds. The final survival gap between the youngest and the oldest patients was about 40%. The 1-year survival figures from NORDCAN are shown in Table S2. The best survival is underlined, dominated by SE, while DK never had the best figures. The bottom row shows combined data for all ages; in the last period, it varied from 81.5% for DK to 88.8% for SE. All age-specific survival figures increased significantly (i.e., non-overlapping CIs) throughout the 50-year period, except those for the FI and NO 80–89-year-olds.

**Figure 2:**
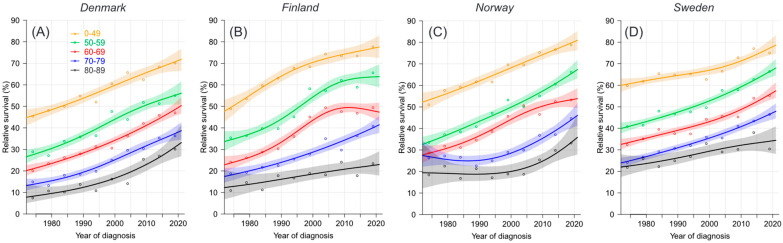
Relative 5-year survival in age groups with ovarian cancer in Denmark (**A**), Finland (**B**), Norway (**C**) and Sweden (**D**), 1972–1976 to 2017–2021. Data were modelled based on the NORDCAN database.

For 5-year survival, the starting levels were around 20% lower than those for 1-year survival, but the curves increased in a linear or modestly superlinear course. However, in FI, the curve for the 60–69-year-old group culminated before the year 2010, and even for the younger patients, the increase slowed down (Figure 2). In NO and SE, the youngest patients reached an 80% survival, while the next age group was 10 units lower, followed by the next age group also 10 units lower. In NO and SE, 80–89-year-old patients reached a 35% survival rate. In DK and FI, survival among patients younger than 60 years was close to the NO/SE level, but fell behind for the older patients; in FI, the 80–89-year-old group barely reached a 20% survival rate. The 5-year survival figures from NORDCAN are shown in Table S3. The best survival is underlined, and these are dominated by SE. The final combined SE survival of 53.2% was significantly higher than survival in DK (45.5%) and in FI (49.1%). Moreover, the final survival in SE some age groups significantly exceeded the DK figures. All age-specific survival figures increased significantly throughout the 50-year period, except those for the FI, NO and SE 80–89-year-olds.

The data in Table S3 can be used to calculate the differences in survival between the youngest and oldest age groups in 1972–1976 and 2017–2021. For DK, there was an increase from 38.0% to 40.0% (a difference of 2.0%); in FI, 37.9% to 54.3% (a difference of 16.4%); in NO, 32.6% to 45.6% (a difference of 13.0%); and in SE, 38.0% to 44.6% (a difference of 6.6%). Thus, the periodic survival gap widened in each country for 5-year survival with an average of 9.5%.

**Figure 3:**
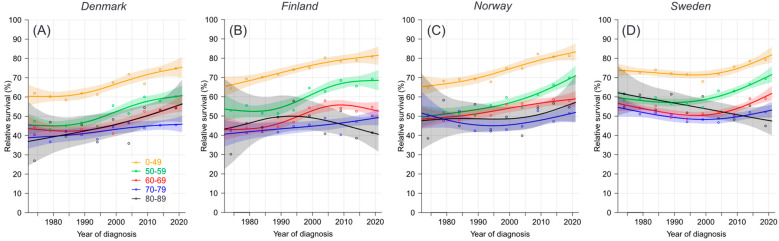
Relative 5/1-year survival in age groups with ovarian cancer in Denmark (**A**), Finland (**B**), Norway (**C**) and Sweden (**D**), 1972–1976 to 2017–2021. Data were modelled based on the NORDCAN database.

Conditional 5/1-year survival curves for ovarian cancer increased only for patients (least in SE) below 60 years (Figure 3). For the older patients, the increase started later, before the year 2000; for SE, the increase started around 2010. The exact age-specific 5/1-year survival figures are shown in Table 1. The overall survival (bottom row) increased significantly by about 10% in all countries except SE. However, significant increases were found for only one or two of the younger age groups.

The relative 5-year survival in the US SEER database for the years 2013–2019 among White (including Hispanics) women was 50.3%; according to age groups, the survival was 82.3% (15–39 years), 59.9% (40–64 years), 42.6% (65–74 years) and 25.0% (75+ years).

### 3.3. Age-Specific Mortality

**Figure 4:**
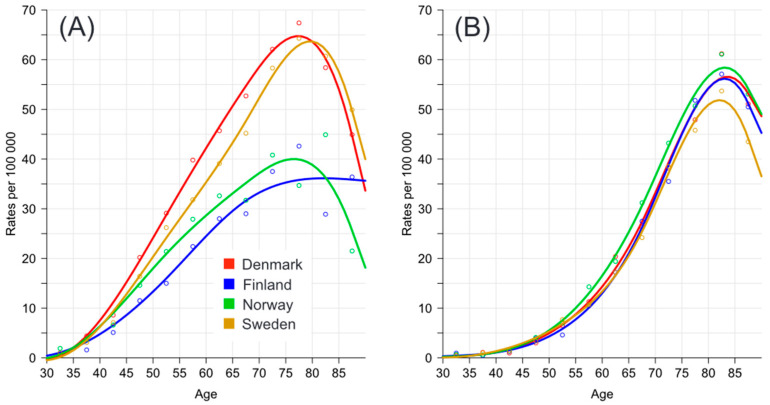
Age-specific mortality in the Nordic countries in patients with ovarian cancer in 1972–1976 (**A**) and in 2027–2021 (**B**). NORDCAN data were interpolated by smoothing splines. The oldest age group was 85+ years old.

Age-specific mortality was analyzed in two periods, at the beginning (1972–1976) and the end (2017–2021) of the study (Figure 4). A major shift had taken place; in the early period, DK and SE were the high mortality countries, while FI and NO were the low mortality countries, all with broad peaks; conversely, in the last period, mortality profiles were unified into sharp peaks culminating at around 82 years. Much of the early age mortality had disappeared with time.

## 4. Discussion

This study reported large age differences in ovarian cancer survival, with an increasing age disadvantage for the old patients over the 50 year time period. The unique novel results on ovarian cancer were revealed by the conditional survival analysis, which documented no increase in survival for the elderly population in the 50-year period from the first year of their diagnosis through four subsequent years. This is a surprising finding, hidden by the large increase in 1-year survival for all age groups, which masked the poor development after year 1. This kind of uneven improvement for survival offers an excellent justification for conditional survival analysis. Similar conclusions of no long-term survival improvement were reached by analysing 10-year survival in the Netherlands, and later in Sweden [7,20]. Another way of estimating periodic differences in survival is to simply compare the differences between 1- and 5-year survival at different times. For many fatal cancers, such as pancreatic and esophageal cancer, which are often diagnosed at the metastatic stage, 1-year survival has improved more than 5-year survival [6]. The likely reason for this is the extension of survival past year 1 without being able to help the patients much further. In fact, this is exactly how the above Dutch study described the survival landscape for ovarian cancer: “The observed improvements in 5-year overall survival reflect a more prolonged disease control rather than better chance for survival” [7].

In agreement with the above statement, and the fact that the results from conditional survival were uniform in the four Nordic countries, we hypothesize that treatment changes modified survival; further information can be gained by looking into the development of treatment for ovarian cancer. The role of cytoreductive surgery was detected early, and some clinics were already applying cytoreduction for all primary ovarian cancers in combination with chemotherapy with agents such as cisplatin, cyclophosphamide and vinblastine in the 1980s [21]. Successful cytoreduction has been reported to double the median survival of ovarian cancer patients, and this has been repeatedly confirmed [22,23,24]. In Sweden, regional clinical guidelines were established in the early 1990s (and national guidelines in 2012); almost all patients underwent surgery, and most also had chemotherapy with carboplatin-based drugs [25]. The guidelines emphasized the creation of surgical teams and the concentration of treatment to fewer centers, in agreement with similar restructuring in the other Nordic countries [24]. The first ESMO minimum clinical recommendations for ovarian cancer, published in 2001, recommended surgery for all FIGO stages, with maximal cytoreduction for the higher stages: chemotherapy with carboplatin (or cisplatin) and paclitaxel was recommended for all stages = In recent ESMO clinical practice guidelines, patients of low-risk FIGO stage I may be observed and those of higher risk stages I and II should receive carboplatin–paclitaxel chemotherapy [3]. Stage III and IV patients are first evaluated for the likelihood of complete cytoreduction, and based on the results, may either receive neoadjuvant chemotherapy followed by interval cytoreduction, or undergo primary cytoreduction followed by standard chemotherapy, or depending on BRCA mutation status (or homologous recombination deficiency for other reasons), PARP inhibitors (olaparib) or antiangiogenic therapy (bevacizumab) or both [3]. As up to 70% of stage III and IV patients will relapse within 3 years, further management guidelines were presented for these patients. Patient age (apart from fertility preservation) is not a theme in these guidelines, except indirectly by reference to feasibility of therapy and performance status [1]. The strong improvement in 1-year survival that we observed can probably be largely explained by earlier diagnosis because of improved imaging, more active and centralized treatment, and optimal palliative care, shifting the survival of incurable high-stage patients past year 1.

Age differences in ovarian cancer survival have been reported in earlier studies, including the recent DK and SE studies [20,24]. The DK study discussed the evidence and the theories for the age disparity, considering limited offered treatment, toleration of treatment, late stage disease at presentation, tumor biological differences and comorbidities [24]. The worrisome findings of the present study documented the widening age gap for each country. The mortality data comparing the early 1972–1976 and late 2017–2021 periods showed a marked shift of mortality ages towards higher ages, with a concomitant large reduction of early onset mortality. This would be in line with the notion that the offered therapies preferentially reduced mortality in younger patients who also tolerate chemotherapy and radical surgery better than the older patients.

In the US SEER database, ovarian cancer 5-year survival for White women (including Hispanics) for the years 2013–2019 was 50.3%, below NO (53.0%) and SE (53.2%), but over DK and FI. The age gradient appeared to be quite steep in the USA, as the 5-year survival for 75+ year-olds was only 25.0%, which was lower than the present survival for 80–89-year-olds (except in FI).

The limitations in the NORDCAN database include the lack of data regarding clinical presentation and treatment. However, the unique advantages of these data are their long follow-time from high-level cancer registries, with long-term collaboration helping to unify the registration practices. Such long-term national data are unavailable outside the Nordic countries.

## 5. Conclusions

We demonstrated strong age-dependence and widening age disparity in ovarian cancer survival in the Nordic countries. Improvement in 1-year survival was very strong, to the extent that it masked the meager development between years 1 and 5 after diagnosis. The modest or lack of improvement in this time interval was revealed by exploring the conditional 5/1-year survival, which improved only for the young age groups. The current treatment practices, which appear increasingly effective for younger patients, have not helped remedy the large age differences in survival. Early detection methods, novel targeted therapies and therapeutic innovations, such as the still experimental, hyperthermic intraperitoneal chemotherapy (HIPEC), are needed [26]. Most ovarian cancer patients present at an advanced stage, for which surgery combined with platinum based chemotherapy, together with PARP inhibition and bevacizumab, have provided increased progression-free and overall survival rates. A key factor in the utility of these intensive therapies is the general health of the patient, which correlates with age and comorbidities. Of note, platinum resistance emerging through the use of chemotherapy, as well as platinum refractory disease, continue to lack effective therapies. Elahere was recently approved for the treatment of folate receptor alpha-positive, platinum-resistant ovarian cancer. Nonetheless, many chemotherapies are used in platinum resistant disease, probably prolonging survival, but the feasibility of these therapies declines with the physical and biological age of patients, perhaps explaining some of the large age disparity in survival. Thus, better tolerated therapies, perhaps with alternative mechanisms of action, would be vital to increase the survival of older (and younger) patients.

## Figures and Tables

**Table 1:** Age-specific 5/1-year conditional relative survival (Pohar Perme estimates [95% CI]) in ovarian cancer in the Nordic countries (1972–2021). The asterisk marks a significant increase in the last period compared to the first period.

**Denmark**										
Age group	**1972–1976**	**1977–1981**	**1982–1986**	**1987–1991**	**1992–1996**	**1997–2001**	**2002–2006**	**2007–2011**	**2012–2016**	**2017–2021**
0–49	61.9 [57.1–67.2]	60.2 [55.3–65.5]	58.5 [53.8–63.7]	61.9 [57.8–66.4]	61.2 [56.8–66.1]	67.5 [63.3–72.1]	71.9 [67.2–76.9]	66.9 [61.9–72.4]	74.3 [69.4–79.6]	74.8 [69.8–80.2] *
50–59	47.4 [42.5–52.8]	43.2 [38.6–48.2]	45.1 [40.6–50.2]	46.6 [42.1–51.6]	44.8 [40.7–49.4]	55.5 [51.7–59.7]	51.4 [47.2–55.9]	60.0 [55.6–64.8]	57.8 [53.1–63.0]	60.2 [55.4–65.5]*
60–69	44.4 [38.8–50.7]	42.5 [37.6–48.0]	41.9 [37.6–46.7]	43.1 [38.6–48.1]	44.7 [40.5–49.2]	41.0 [36.9–45.6]	46.5 [42.4–50.9]	52.6 [48.7–56.7]	52.8 [48.9–57.0]	54.6 [50.7–58.8]
70–79	40.5 [33.0–49.7]	36.8 [30.3–44.6]	42.3 [36.1–49.5]	40.4 [34.3–47.5]	38.1 [32.7–44.3]	44.5 [39.2–50.5]	46.5 [41.3–52.4]	43.9 [39.1–49.2]	45.3 [41.1–49.9]	45.5 [41.4–50.0] *
80–89	26.9 [13.0–55.7]	46.9 [30.4–72.3]	39.5 [25.6–61.1]	45.5 [32.0–64.7]	36.6 [24.1–55.7]	48.4 [34.9–67.0]	35.9 [25.4–50.7]	54.5 [44.0–67.6]	53.3 [43.1–65.8]	54.2 [44.1–66.7]
Age stand	45.9 [42.7–49.3]	45.1 [42.3–48.1]	45.6 [43.0–48.5]	46.9 [44.3–49.5]	45.4 [42.9–47.9]	49.9 [47.5–52.4]	50.6 [48.1–53.1]	54.1 [51.7–56.6]	54.7 [52.4–57.1]	55.8 [53.5–58.3]*
**Finland**										
Age group	**1972–1976**	**1977–1981**	**1982–1986**	**1987–1991**	**1992–1996**	**1997–2001**	**2002–2006**	**2007–2011**	**2012–2016**	**2017–2021**
0–49	64.4 [58.6–70.8]	69.2 [63.7–75.1]	70.2 [65.2–75.5]	71.5 [67.0–76.4]	74.1 [70.1–78.4]	75.1 [70.8–79.6]	80.2 [76.1–84.6]	78.4 [73.7–83.5]	78.9 [74.1–84.0]	80.9 [76.2–85.9] *
50–59	53.0 [47.0–59.7]	55.5 [49.6–62.0]	51.4 [46.1–57.2]	51.3 [46.0–57.1]	53.0 [48.1–58.4]	64.5 [60.3–69.0]	62.8 [58.8–67.0]	68.5 [64.2–73.0]	65.5 [60.9–70.5]	69.1 [64.4–74.2] *
60–69	43.9 [38.0–50.7]	43.9 [38.4–50.2]	42.6 [37.4–48.6]	44.5 [39.7–49.8]	46.8 [42.5–51.6]	54.3 [49.8–59.1]	57.9 [53.8–62.3]	54.1 [50.3–58.2]	52.5 [48.6–56.6]	54.6 [50.7–58.7]
70–79	42.5 [34.0–53.2]	41.3 [33.9–50.3]	41.7 [34.8–50.0]	41.6 [35.3–49.0]	44.7 [38.4–52.0]	45.4 [39.7–52.0]	49.0 [43.7–54.8]	40.2 [35.4–45.7]	47.1 [42.5–52.1]	50.1 [45.6–55.0]
80–89	30.2 [11.3–80.6]	46.2 [29.1–73.2]	44.1 [26.6–73.1]	49.4 [33.8–72.4]	57.8 [41.0–81.5]	50.1 [36.3–69.2]	40.8 [30.5–54.6]	52.7 [42.3–65.7]	38.5 [29.8–49.8]	41.3 [32.3–52.9]
Age stand	47.8 [43.6–52.3]	50.2 [46.8–53.8]	48.9 [45.9–52.1]	50.2 [47.3–53.2]	52.8 [50.0–55.7]	57.1 [54.6–59.8]	58.2 [56.0–60.6]	56.4 [54.3–58.6]	55.9 [53.7–58.1]	58.3 [56.1–60.6] *
**Norway**										
Age group	**1972–1976**	**1977–1981**	**1982–1986**	**1987–1991**	**1992–1996**	**1997–2001**	**2002–2006**	**2007–2011**	**2012–2016**	**2017–2021**
0–49	64.8 [59.3–70.8]	68.2 [63.3–73.6]	69.1 [64.3–74.2]	69.2 [64.8–74.0]	67.8 [63.4–72.6]	74.9 [70.8–79.3]	74.7 [70.3–79.4]	82.3 [78.0–86.8]	80.8 [76.4–85.4]	81.3 [77.0–85.9] *
50–59	50.9 [45.2–57.4]	52.0 [46.6–57.9]	51.2 [46.2–56.8]	50.6 [45.4–56.5]	55.6 [50.8–60.9]	59.7 [55.3–64.4]	54.5 [50.3–59.1]	61.2 [56.8–65.8]	66.2 [62.0–70.8]	69.8 [65.5–74.5] *
60–69	48.8 [42.8–55.7]	51.7 [46.2–57.8]	48.0 [43.1–53.5]	50.4 [45.5–55.8]	50.3 [45.3–55.9]	56.1 [51.2–61.5]	56.5 [52.0–61.5]	55.2 [51.1–59.5]	58.6 [54.6–62.8]	58.1 [54.1–62.4]
70–79	50.2 [41.5–60.7]	47.8 [40.3–56.7]	52.7 [45.5–60.9]	42.4 [36.2–49.6]	43.3 [37.5–49.9]	43.0 [38.0–48.7]	44.8 [39.2–51.1]	52.8 [47.0–59.3]	47.3 [42.0–53.1]	51.4 [46.5–56.9]
80–89	38.5 [21.7–68.2]	58.3 [38.6–88.0]	44.9 [29.8–67.6]	56.2 [42.8–73.8]	42.2 [30.1–59.2]	49.6 [36.9–66.7]	39.9 [31.0–51.3]	54.2 [43.6–67.3]	56.7 [46.1–69.7]	54.6 [43.9–68.0]
Age stand	50.9 [46.9–55.1]	54.1 [50.4–58.0]	53.1 [50.0–56.5]	52.3 [49.4–55.3]	51.7 [49.2–54.3]	55.6 [53.1–58.3]	54.3 [51.9–56.9]	59.5 [57.1–62.0]	59.8 [57.4–62.3]	61.1 [58.7–63.7] *
**Sweden**										
Age group	**1972–1976**	**1977–1981**	**1982–1986**	**1987–1991**	**1992–1996**	**1997–2001**	**2002–2006**	**2007–2011**	**2012–2016**	**2017–2021**
0–49	73.5 [70.4–76.7]	72.6 [69.5–75.9]	74.0 [70.8–77.3]	72.1 [68.8–75.6]	71.6 [68.2–75.2]	68.0 [64.4–71.8]	71.9 [68.3–75.7]	75.7 [71.9–79.7]	78.6 [74.9–82.4]	79.3 [75.6–83.1]
50–59	58.2 [55.0–61.6]	59.0 [55.6–62.6]	59.6 [56.2–63.2]	56.6 [53.0–60.5]	56.1 [52.6–59.8]	54.5 [51.3–58.0]	63.2 [59.8–66.7]	63.0 [59.3–66.9]	66.6 [62.9–70.4]	69.5 [65.8–73.4] *
60–69	55.0 [51.3–59.0]	54.2 [50.7–57.8]	55.2 [52.2–58.4]	49.2 [46.0–52.7]	48.5 [45.1–52.2]	51.3 [47.8–55.0]	52.2 [48.9–55.6]	51.0 [47.9–54.3]	59.1 [56.0–62.3]	59.1 [55.8–62.6]
70–79	54.6 [49.4–60.5]	51.1 [46.3–56.3]	51.7 [47.3–56.5]	50.7 [46.5–55.2]	47.1 [43.3–51.1]	48.3 [44.4–52.4]	46.7 [42.4–51.4]	50.2 [46.2–54.5]	52.0 [48.1–56.2]	52.1 [48.4–56.0]
80–89	61.6 [47.8–79.3]	61.1 [48.5–76.9]	53.2 [42.1–67.3]	61.3 [50.5–74.5]	51.7 [42.1–63.6]	60.3 [50.5–71.8]	48.8 [41.1–57.9]	48.2 [40.4–57.6]	55.4 [46.6–65.9]	44.9 [36.8–54.8]
Age stand	59.4 [57.0–61.9]	58.2 [55.8–60.6]	58.2 [56.1–60.3]	55.7 [53.7–57.8]	53.3 [51.4–55.3]	54.3 [52.4–56.3]	55.1 [53.2–57.1]	56.2 [54.2–58.2]	60.8 [58.7–62.9]	59.9 [58.0–61.8]

## Data Availability

A publicly available database was used.

## Supplementary Materials

The following supporting information can be downloaded at: https://www.mdpi.com/article/10.3390/cancers16122198/s1, Table S1. Case numbers and estimated median ages at diagnosis for ovarian cancer in the Nordic countries in 1972–1976 and 2017–2021 based on the NORDCAN data; Table S2. Age-specific 1-year relative survival (Pohar Perme estimates [95% CI]) in ovarian cancer in the Nordic countries (1972–2021). The best age-specific relative survival for each period is underlined. The asterisk marks a significant increase between the first and the last period; Table S3. Age-specific 5-year relative survival (Pohar Perme estimates [95% CI]) in ovarian cancer in the Nordic countries (1972–2021). The best age-specific relative survival for each period is underlined. The asterisk marks a significant increase between the first and the last period.

## References

[1] Ledermann J.A., Matias-Guiu X., Amant F., Concin N., Davidson B., Fotopoulou C., González-Martin A., Gourley C., Leary A., Lorusso D. (2024). ESGO-ESMO-ESP consensus conference recommendations on ovarian cancer: Pathology and molecular biology and early, advanced and recurrent disease. Ann. Oncol..

[2] Tichanek F., Försti A., Hemminki O., Hemminki A., Hemminki K. (2023). Survival, Incidence, and Mortality Trends in Female Cancers in the Nordic Countries. Obstet. Gynecol. Int..

[3] González-Martín A., Harter P., Leary A., Lorusso D., Miller R.E., Pothuri B., Ray-Coquard I., Tan D.S.P., Bellet E., Oaknin A. (2023). Newly diagnosed and relapsed epithelial ovarian cancer: ESMO Clinical Practice Guideline for diagnosis, treatment and follow-up. Ann. Oncol..

[4] Hemminki K., Sundquist K., Sundquist J., Försti A., Hemminki A., Li X. (2021). Familial Risks and Proportions Describing Population Landscape of Familial Cancer. Cancers.

[5] Ledermann J.A., Raja F.A., Fotopoulou C., Gonzalez-Martin A., Colombo N., Sessa C. (2013). Newly diagnosed and relapsed epithelial ovarian carcinoma: ESMO Clinical Practice Guidelines for diagnosis, treatment and follow-up. Ann. Oncol..

[6] Hemminki J., Försti A., Hemminki A., Hemminki K. (2022). Survival trends in solid cancers in the Nordic countries through 50 years. Eur. J. Cancer.

[7] Timmermans M., Sonke G.S., Van de Vijver K.K., van der Aa M.A., Kruitwagen R. (2018). No improvement in long-term survival for epithelial ovarian cancer patients: A population-based study between 1989 and 2014 in the Netherlands. Eur. J. Cancer.

[8] Allemani C., Matsuda T., Di Carlo V., Harewood R., Matz M., Nikšić M., Bonaventure A., Valkov M., Johnson C.J., Estève J. (2018). Global surveillance of trends in cancer survival 2000-14 (CONCORD-3): Analysis of individual records for 37 513 025 patients diagnosed with one of 18 cancers from 322 population-based registries in 71 countries. Lancet.

[9] Yun B.S., Park E.H., Ha J., Lee J.Y., Lee K.H., Lee T.S., Lee K.J., Kim Y.J., Jung K.W., Roh J.W. (2023). Incidence and survival of gynecologic cancer including cervical, uterine, ovarian, vaginal, vulvar cancer and gestational trophoblastic neoplasia in Korea, 1999–2019: Korea Central Cancer Registry. Obstet. Gynecol. Sci..

[10] Pukkala E., Engholm G., Højsgaard S.L.K., Storm H., Khan S., Lambe M., Pettersson D., Ólafsdóttir E., Tryggvadóttir L., Hakanen T. (2018). Nordic Cancer Registries—An overview of their procedures and data comparability. Acta Oncol..

[11] Engholm G., Ferlay J., Christensen N., Bray F., Gjerstorff M.L., Klint A., Køtlum J.E., Olafsdóttir E., Pukkala E., Storm H.H. (2010). NORDCAN—A Nordic tool for cancer information, planning, quality control and research. Acta Oncol..

[12] Larønningen S., Arvidsson G., Bray F., Engholm G., Ervik M., Guðmundsdóttir E.M., Gulbrandsen J., Hansen H.L., Hansen H.M., Johannesen T.B. (2023). NORDCAN: Cancer Incidence, Mortality, Prevalence and Survival in the Nordic Countries, Version 9.3. https://nordcan.iarc.fr/en/database.

[13] Storm H.H., Klint A., Tryggvadóttir L., Gislum M., Engholm G., Bray F., Hakulinen T. (2010). Trends in the survival of patients diagnosed with malignant neoplasms of lymphoid, haematopoietic, and related tissue in the Nordic countries 1964–2003 followed up to the end of 2006. Acta Oncol..

[14] Engholm G., Gislum M., Bray F., Hakulinen T. (2010). Trends in the survival of patients diagnosed with cancer in the Nordic countries 1964–2003 followed up to the end of 2006. Material and methods. Acta Oncol..

[15] Lundberg F.E., Ekman S., Johansson A.L.V., Engholm G., Birgisson H., Ólafsdóttir E.J., Mørch L.S., Johannesen T.B., Andersson T.M., Pettersson D. (2020). Trends in cancer survival in the Nordic countries 1990–2016: The NORDCAN survival studies. Acta Oncol..

[16] Tichanek F., Försti A., Liska V., Hemminki A., Hemminki K. (2023). Survival in Colon, Rectal and Small Intestinal Cancers in the Nordic Countries through a Half Century. Cancers.

[17] Bürkner P. (2017). An R package for Bayesian multilevel models using Stan. J. Stat. Softw..

[18] Bürkner P. (2018). Advanced Bayesian multilevel modeling with the R package (brms). R J..

[19] Carpenter B., Gelman A., Hoffman M.D., Lee D., Goodrich B., Betancourt M., Brubaker M., Guo J., Li P., Riddell A. (2017). Stan: A probabilistic programming language. J. Stat. Softw..

[20] Leandersson P., Hogberg T., Dickman P.W., Malander S., Borgfeldt C. (2021). Incidence and survival of epithelial ovarian, fallopian tube, peritoneal, and undesignated abdominal/pelvic cancers in Sweden 1960–2014: A population-based cohort study. BMC Cancer.

[21] Berek J.S., Hacker N.F., Lagasse L.D. (1982). Recent progress in the treatment of epithelial ovarian malignancy. West. J. Med..

[22] Fader A.N., Rose P.G. (2007). Role of surgery in ovarian carcinoma. J. Clin. Oncol..

[23] Fortner R.T., Trewin-Nybråten C.B., Paulsen T., Langseth H. (2023). Characterization of ovarian cancer survival by histotype and stage: A nationwide study in Norway. Int. J. Cancer.

[24] Mallen A., Todd S., Robertson S.E., Kim J., Sehovic M., Wenham R.M., Extermann M., Chon H.S. (2021). Impact of age, comorbidity, and treatment characteristics on survival in older women with advanced high grade epithelial ovarian cancer. Gynecol. Oncol..

[25] Akeson M., Zetterqvist B.M., Holmberg E., Horvath G. (2005). Improved survival with clinical guidelines? Evaluation of a quality register linked to clinical guidelines for ovarian cancer in the western health care region in Sweden between 1 September 1993 and 1 June 1998. Acta Obstet. Gynecol. Scand..

[26] Aronson S.L., Lopez-Yurda M., Koole S.N., Schagen van Leeuwen J.H., Schreuder H.W.R., Hermans R.H.M., de Hingh I.H.J.T., van Gent M.D.J.M., Arts H.J.G., van Ham M.A.P.C. (2023). Cytoreductive surgery with or without hyperthermic intraperitoneal chemotherapy in patients with advanced ovarian cancer (OVHIPEC-1): Final survival analysis of a randomised, controlled, phase 3 trial. Lancet Oncol..

