# Supplementary material for: Survival in Elderly Ovarian Cancer Remains Challenging in the Nordic Countries
Source: Cancers (Basel). 2024 Jun 11;16(12):2198. doi: 10.3390/cancers16122198 (PMC11201377; doi:10.3390/cancers16122198)
Supplement: Supplementary file 1 [file cancers-16-02198-s001.zip › cancers-3019747-supplementary.pdf]

Supplementary Table S1. Case numbers and estimated median ages at diagnosis for ovarian cancer in the Nordic countries in 1972-1976 and 2017-2021 based on the NORDCAN data.

	**1972-1976**		**2017-2021**	
	**N**	**median age**	**N**	**median age**
Denmark	2873	63.2	2753	68.8
Finland	1586	60.5	2735	69.6
Norway	1629	61.6	2159	67.2
Sweden	5083	62.7	3473	67.3
**Total**	**11171**	**62.4**	**11138**	**68.2**

Supplementary Table S2. Age-specific 1-year relative survival (Pohar Perme estimates [95% CI]) in ovarian cancer in the Nordic countries (1972-2021). The best age-specific relative survival for each period is underlined. The asterisk marks significant increase between the first and the last period.

Denmark

Age group	**1972-1976**	**1977-1981**	**1982-1986**	**1987-1991**	**1992-1996**	**1997-2001**	**2002-2006**	**2007-2011**	**2012-2016**	**2017-2021**
0-49	73.3 [69.5 - 77.3]	79.9 [76.3 - 83.7]	85.4 [82.3 - 88.6]	88.5 [85.9 - 91.2]	84.9 [81.8 - 88.1]	89.6 [86.9 - 92.4]	91.4 [88.6 - 94.3]	93.1 [90.4 - 95.9]	91.9 [88.9 - 95.0]	93.7 [91.0 - 96.5]*
50-59	61.0 [57.3 - 64.9]	62.8 [59.2 - 66.6]	74.9 [71.5 - 78.5]	76.8 [73.3 - 80.5]	81.2 [78.1 - 84.4]	85.7 [83.1 - 88.4]	85.4 [82.7 - 88.2]	86.3 [83.4 - 89.3]	88.7 [85.8 - 91.7]	91.3 [88.6 - 94.1]*
60-69	45.3 [41.8 - 49.1]	55.1 [51.7 - 58.7]	62.5 [59.2 - 66.0]	64.7 [61.4 - 68.2]	70.1 [66.9 - 73.5]	74.6 [71.5 - 77.8]	77.9 [75.0 - 80.9]	80.1 [77.3 - 83.0]	86.7 [84.3 - 89.2]	86.1 [83.5 - 88.8]*
70-79	36.8 [32.9 - 41.2]	35.9 [32.3 - 39.9]	42.6 [38.9 - 46.7]	45.6 [41.8 - 49.7]	52.0 [48.2 - 56.1]	56.2 [52.7 - 59.9]	63.6 [59.9 - 67.5]	69.3 [65.8 - 73.0]	77.9 [74.9 - 81.0]	79.8 [77.0 - 82.7]*
80-89	27.5 [20.9 - 36.2]	22.8 [17.8 - 29.2]	25.8 [21.0 - 31.7]	30.1 [25.0 - 36.2]	29.5 [24.3 - 35.8]	33.5 [28.2 - 39.8]	39.3 [34.0 - 45.4]	46.6 [41.5 - 52.3]	50.5 [45.2 - 56.4]	55.5 [50.1 - 61.5]*
**Age stan.**	**46.2 [44.2 - 48.3]**	**49.0 [47.2 - 50.9]**	**56.1 [54.3 - 57.9]**	**58.9 [57.1 - 60.7]**	**62.4 [60.6 - 64.2]**	**66.8 [65.1 - 68.5]**	**70.8 [69.2 -72.6]**	**74.5 [72.9 -76.2]**	**79.7 [78.2 - 81.2]**	**81.5 [80.0 - 83.0]***

Finland

Age group	**1972-1976**	**1977-1981**	**1982-1986**	**1987-1991**	**1992-1996**	**1997-2001**	**2002-2006**	**2007-2011**	**2012-2016**	**2017-2021**
0-49	75.8 [71.3 - 80.6]	77.3 [73.0 - 81.9]	85.1 [81.5 - 88.9]	88.2 [85.0 - 91.5]	91.5 [89.0 - 94.1]	91.1 [88.5 - 93.8]	92.6 [90.0 - 95.3]	93.6 [90.9 - 96.4]	93.0 [90.0 - 96.1]	96.0 [93.9 - 98.1]*
50-59	66.8 [62.2 - 71.7]	65.6 [61.1 - 70.4]	77.3 [73.4 - 81.4]	77.4 [73.4 - 81.6]	85.1 [81.8 - 88.5]	90.2 [87.7 - 92.8]	91.3 [88.9 - 93.8]	90.7 [88.2 - 93.3]	89.9 [87.1 - 92.8]	94.9 [92.6 - 97.3]*
60-69	54.9 [50.5 - 59.7]	59.4 [55.2 - 63.9]	67.6 [63.5 - 72.0]	68.1 [64.4 - 72.0]	76.9 [73.7 - 80.2]	83.1 [80.1 - 86.2]	85.3 [82.6 - 88.1]	88.0 [85.6 - 90.5]	89.2 [87.0 - 91.5]	90.5 [88.2 - 92.9]*
70-79	44.0 [38.5 - 50.3]	46.7 [41.8 - 52.2]	52.0 [47.4 - 57.0]	54.1 [49.7 - 58.9]	57.1 [52.8 - 61.8]	61.2 [57.2 - 65.5]	71.5 [67.9 - 75.3]	73.8 [70.3 - 77.5]	77.1 [73.9 - 80.4]	81.5 [78.7 - 84.4]*
80-89	36.1 [24.6 - 53.0]	31.6 [23.8 - 42.0]	25.4 [19.3 - 33.4]	36.0 [29.6 - 43.8]	28.9 [23.6 - 35.4]	37.7 [31.7 - 44.8]	44.6 [39.0 - 51.0]	45.7 [40.3 - 51.8]	46.2 [41.0 - 52.1]	56.6 [51.0 - 62.8]
**Age stan.**	**53.4[50.4 - 56.6]**	**54.8 [52.3 - 57.3]**	**60.5 [58.3 - 62.8]**	**63.2 [61.1 - 65.4]**	**67.1 [65.2 - 69.1]**	**72.1 [70.3 - 74.0]**	**77.1 [75.4 - 78.8]**	**78.7 [77.1 - 80.3]**	**79.8 [78.3 - 81.4]**	**84.2 [82.8 - 85.7]***

Norway

Age group	**1972-1976**	**1977-1981**	**1982-1986**	**1987-1991**	**1992-1996**	**1997-2001**	**2002-2006**	**2007-2011**	**2012-2016**	**2017-2021**
0-49	78.7 [74.6 - 83.0]	84.4 [80.9 - 88.1]	85.7 [82.3 - 89.2]	89.1 [86.2 - 92.1]	90.8 [88.2 - 93.5]	92.9 [90.5 - 95.4]	93.0 [90.4 - 95.7]	91.5 [88.5 - 94.6]	94.8 [92.3 - 97.4]	96.9 [94.9 - 98.9]*
50-59	65.8 [61.5 - 70.4]	71.4 [67.3 - 75.7]	75.0 [71.2 - 79.0]	80.6 [76.8 - 84.6]	84.7 [81.5 - 88.0]	89.1 [86.4 - 91.9]	91.9 [89.5 - 94.4]	90.1 [87.5 - 92.8]	91.5 [89.1 - 94.0]	94.8 [92.5 - 97.2]*
60-69	58.6 [54.0 - 63.6]	61.5 [57.5 - 65.8]	64.8 [61.1 - 68.7]	68.7 [65.0 - 72.6]	76.7 [73.1 - 80.5]	81.5 [78.1 - 85.0]	89.5 [86.8 - 92.3]	84.3 [81.5 - 87.2]	89.6 [87.2 - 92.1]	91.6 [89.2 - 94.1]*
70-79	52.4 [46.9 - 58.5]	56.9 [51.8 - 62.5]	50.5 [46.1 - 55.3]	53.3 [49.0 - 58.0]	57.8 [53.8 - 62.1]	67.2 [63.5 - 71.1]	66.8 [62.7 - 71.2]	69.7 [65.6 - 74.1]	78.5 [74.9 - 82.3]	86.7 [83.8 - 89.7]*
80-89	47.8 [37.6 - 60.8]	38.6 [30.1 - 49.5]	37.4 [30.9 - 45.3]	37.9 [32.2 - 44.6]	40.5 [34.7 - 47.3]	37.9 [32.2 - 44.6]	46.9 [41.9 - 52.5]	46.9 [41.5 - 53.0]	52.6 [46.8 - 59.1]	60.8 [54.1 - 68.3]
**Age stan.**	**58.8 [56.0 - 61.8]**	**61.2 [58.7 -63.8]**	**60.8 [58.7 - 63.0]**	**64.1 [62.1 - 66.1]**	**68.9 [66.9 - 70.8]**	**73.5 [71.7 - 75.3]**	**77.3 [75.6 - 79.1]**	**76.3 [74.5 - 78.1]**	**81.8 [80.1 - 83.4]**	**86.7 [85.2 - 88.3]***

Sweden

Age group	**1972-1976**	**1977-1981**	**1982-1986**	**1987-1991**	**1992-1996**	**1997-2001**	**2002-2006**	**2007-2011**	**2012-2016**	**2017-2021**
0-49	81.4 [78.9 - 84.0]	85.1 [82.8 - 87.5]	88.4 [86.2 - 90.7]	89.9 [87.9 - 91.9]	91.0 [89.0 - 93.0]	92.2 [90.2 - 94.2]	92.5 [90.3 - 94.8]	96.2 [94.4 - 98.0]	98.0 [96.7 - 99.3]	94.6 [92.6 - 96.6]*
50-59	70.1 [67.5 - 72.8]	70.2 [67.6 - 72.9]	80.5 [78.0 - 83.1]	82.5 [80.0 - 85.1]	84.9 [82.7 - 87.2]	91.0 [89.2 - 92.8]	91.2 [89.3 - 93.1]	91.9 [89.8 - 94.0]	94.2 [92.4 - 96.0]	95.5 [93.9 - 97.1]*
60-69	58.9 [56.3 - 61.6]	64.8 [62.2 - 67.5]	71.5 [69.1 - 74.0]	76.2 [73.9 - 78.6]	77.1 [74.5 - 79.8]	86.2 [83.9 - 88.6]	87.4 [85.4 - 89.4]	88.8 [86.9 - 90.7]	91.4 [89.6 - 93.2]	93.4 [91.7 - 95.1]*
70-79	44.1 [41.1 - 47.3]	52.3 [49.2 - 55.6]	56.3 [53.3 - 59.5]	60.6 [57.7 - 63.6]	68.0 [65.3 - 70.8]	74.4 [71.7 - 77.2]	76.0 [73.0 - 79.1]	81.7 [79.0 - 84.5]	83.5 [81.0 - 86.1]	89.1 [87.1 - 91.1]*
80-89	35.4 [30.3 - 41.4]	42.4 [36.9 - 48.7]	41.7 [36.7 - 47.4]	40.6 [36.0 - 45.8]	51.8 [46.8 - 57.3]	54.6 [49.5 - 60.2]	59.4 [54.8 - 64.4]	62.6 [57.9 - 67.7]	68.6 [63.3 - 74.3]	67.7 [62.4 - 73.5]*
**Age stan.**	**55.7 [54.2 - 57.2]**	**61.2 [59.6 - 62.7]**	**66.2 [64.7 - 67.6]**	**69.1 [67.7 - 70.5]**	**73.7 [72.3 - 75.1]**	**79.6 [78.2 - 80.9]**	**81.1 [79.8 - 82.5]**	**84.2 [83.0 - 85.5]**	**86.9 [85.7 - 88.2]**	**88.8 [87.6 - 90.0]***

Supplementary Table S3. Age-specific 5-year relative survival (Pohar Perme estimates [95% CI]) in ovarian cancer in the Nordic countries (1972-2021). The best age-specific relative survival for each period is underlined. The asterisk marks significant increase between the first and the last period.

Denmark

Age group	**1972-1976**	**1977-1981**	**1982-1986**	**1987-1991**	**1992-1996**	**1997-2001**	**2002-2006**	**2007-2011**	**2012-2016**	**2017-2021**
0-49	45.4 [41.2 - 50.0]	48.1 [43.7 - 52.9]	50.0 [45.6 - 54.8]	54.8 [50.8 - 59.1]	52.0 [47.8 - 56.6]	60.5 [56.3 - 65.0]	65.7 [61.0 - 70.8]	62.3 [57.3 - 67.7]	68.3 [63.3 - 73.7]	70.1 [65.0 - 75.6]*
50-59	28.9 [25.5 - 32.8]	27.1 [23.9 - 30.7]	33.8 [30.1 - 38.0]	35.8 [32.0 - 40.1]	36.4 [32.8 - 40.4]	47.6 [44.0 - 51.5]	43.9 [40.1 - 48.1]	51.8 [47.6 - 56.4]	51.3 [46.8 - 56.2]	55.0 [50.3 - 60.1]*
60-69	20.1 [17.2 - 23.5]	23.4 [20.4 - 26.8]	26.2 [23.2 - 29.6]	27.9 [24.7 - 31.5]	31.3 [28.1 - 34.9]	30.6 [27.3 - 34.3]	36.2 [32.8 - 40.0]	42.1 [38.7 - 45.8]	45.8 [42.2 - 49.7]	47.0 [43.4 - 50.9]*
70-79	14.9 [11.8 - 18.8]	13.2 [10.6 - 16.4]	18.0 [15.0 - 21.6]	18.4 [15.3 - 22.1]	19.8 [16.7 - 23.5]	25.0 [21.7 - 28.8]	29.6 [25.9 - 33.8]	30.4 [26.8 - 34.5]	35.3 [31.8 - 39.2]	36.3 [32.8 - 40.2]*
80-89	7.4 [3.4 - 16.1]	10.7 [6.5 - 17.6]	10.2 [6.3 - 16.5]	13.7 [9.2 - 20.4]	10.8 [6.8 - 17.2]	16.2 [11.2 - 23.4]	14.1 [9.7 - 20.5]	25.4 [19.9 - 32.4]	26.9 [21.2 - 34.1]	30.1 [23.9 - 37.9]*
**Age stand.**	**21.2 [19.5 – 23.0]**	**22.1 [20.5 – 23.9]**	**25.6 [23.9 – 27.4]**	**27.6 [25.9 – 29.4]**	**28.3 [26.6 – 30.1]**	**33.3 [31.5 – 35.2]**	**35.8 [33.9 – 37.8]**	**40.3 [38.3 – 42.3]**	**43.6 [41.6 – 45.6]**	**45.5 [43.4 - 47.6]***

Finland

Age group	**1972-1976**	**1977-1981**	**1982-1986**	**1987-1991**	**1992-1996**	**1997-2001**	**2002-2006**	**2007-2011**	**2012-2016**	**2017-2021**
0-49	48.8 [43.6 - 54.6]	53.5 [48.4 - 59.1]	59.7 [54.8 - 65.0]	63.1 [58.5 - 68.1]	67.8 [63.7 - 72.2]	68.4 [64.1 - 73.0]	74.3 [70.0 - 78.9]	73.4 [68.5 - 78.7]	73.4 [68.4 - 78.8]	77.7 [72.9 - 82.8]*
50-59	35.4 [30.8 - 40.7]	36.4 [31.9 - 41.5]	39.7 [35.2 - 44.8]	39.7 [35.2 - 44.8]	45.1 [40.6 - 50.1]	58.2 [54.1 - 62.6]	57.3 [53.4 - 61.5]	62.1 [57.9 - 66.6]	58.9 [54.4 - 63.8]	65.6 [60.9 - 70.7]*
60-69	24.1 [20.4 - 28.5]	26.1 [22.4 - 30.4]	28.8 [24.9 - 33.3]	30.3 [26.7 - 34.4]	36.0 [32.4 - 40.0]	45.1 [41.1 - 49.5]	49.4 [45.6 - 53.5]	47.6 [44.0 - 51.5]	46.8 [43.2 - 50.7]	49.4 [45.7 - 53.4]*
70-79	18.7 [14.4 - 24.3]	19.3 [15.4 - 24.2]	21.7 [17.7 - 26.6]	22.5 [18.7 - 27.1]	25.5 [21.5 - 30.2]	27.8 [23.9 - 32.3]	35.0 [30.9 - 39.6]	29.7 [25.9 - 34.1]	36.3 [32.5 - 40.5]	40.8 [36.9 - 45.1]*
80-89	10.9 [3.8 - 31.3]	14.6 [8.5 - 25.1]	11.2 [6.3 - 19.9]	17.8 [11.6 - 27.3]	16.7 [11.2 - 24.9]	18.9 [13.1 - 27.3]	18.2 [13.2 - 25.1]	24.1 [18.7 - 31.1]	17.8 [13.4 - 23.6]	23.4 [17.9 - 30.6]
**Age stand.**	**25.5 [22.9 - 28.3]**	**27.5 [25.3 - 29.9]**	**29.6 [27.5 - 31.9]**	**31.7 [29.6 - 34.0]**	**35.4 [33.3 - 37.6]**	**41.2 [39.1 - 43.4]**	**44.9 [42.9 - 47.0]**	**44.4 [42.5 - 46.5]**	**44.6 [42.7 - 46.7]**	**49.1 [47.1 - 51.3]***

Norway

Age group	**1972-1976**	**1977-1981**	**1982-1986**	**1987-1991**	**1992-1996**	**1997-2001**	**2002-2006**	**2007-2011**	**2012-2016**	**2017-2021**
0-49	51.0 [46.0 - 56.5]	57.6 [52.8 - 62.8]	59.2 [54.5 - 64.3]	61.7 [57.3 - 66.4]	61.6 [57.2 - 66.3]	69.6 [65.4 - 74.1]	69.5 [65.0 - 74.3]	75.3 [70.7 - 80.2]	76.6 [72.0 - 81.5]	78.8 [74.3 - 83.6]*
50-59	33.5 [29.2 - 38.4]	37.1 [32.8 - 42.0]	38.4 [34.2 - 43.1]	40.8 [36.2 - 46.0]	47.1 [42.7 - 52.0]	53.2 [49.0 - 57.8]	50.1 [46.0 - 54.6]	55.1 [50.9 - 59.6]	60.6 [56.4 - 65.1]	66.2 [61.8 - 70.9]*
60-69	28.6 [24.5 - 33.4]	31.8 [27.9 - 36.2]	31.1 [27.5 - 35.2]	34.6 [30.8 - 38.9]	38.6 [34.4 - 43.3]	45.7 [41.3 - 50.6]	50.6 [46.3 - 55.3]	46.5 [42.8 - 50.5]	52.5 [48.7 - 56.6]	53.2 [49.3 - 57.4]*
70-79	26.3 [21.1 - 32.8]	27.2 [22.4 - 33.0]	26.6 [22.4 - 31.6]	22.6 [18.9 - 27.0]	25.0 [21.3 - 29.3]	28.9 [25.2 - 33.1]	29.9 [25.8 - 34.7]	36.8 [32.3 - 41.9]	37.1 [32.7 - 42.1]	44.6 [40.1 - 49.6]*
80-89	18.4 [9.9 - 34.2]	22.5 [13.9 - 36.4]	16.8 [10.7 - 26.4]	21.3 [15.5 - 29.3]	17.1 [11.8 - 24.8]	18.8 [13.4 - 26.4]	18.7 [14.2 - 24.6]	25.4 [19.8 - 32.6]	29.8 [23.5 - 37.8]	33.2 [25.9 - 42.6]
**Age stand.**	**29.9 [27.2 - 32.9]**	**33.1 [30.5 - 35.9]**	**32.3 [30.1 - 34.6]**	**33.5 [31.4 - 35.7]**	**35.6 [33.6 - 37.8]**	**40.9 [38.8 - 43.2]**	**42.0 [39.9 - 44.2]**	**45.4 [43.3 -47.7]**	**48.9 [46.7 - 51.2]**	**53.0 [50.7 - 55.4]***

Sweden

Age group	**1972-1976**	**1977-1981**	**1982-1986**	**1987-1991**	**1992-1996**	**1997-2001**	**2002-2006**	**2007-2011**	**2012-2016**	**2017-2021**
0-49	59.8 [56.7 - 63.1]	61.8 [58.7 - 65.1]	65.4 [62.2 - 68.8]	64.8 [61.5 - 68.3]	65.2 [61.8 - 68.8]	62.7 [59.1 - 66.5]	66.5 [62.8 - 70.4]	72.8 [68.9 - 76.9]	77.0 [73.3 - 80.9]	75.0 [71.2 - 79.0]*
50-59	40.8 [38.1 - 43.7]	41.4 [38.6 - 44.4]	48.0 [44.9 - 51.3]	46.7 [43.4 - 50.3]	47.6 [44.4 - 51.0]	49.6 [46.5 - 52.9]	57.6 [54.3 - 61.1]	57.9 [54.3 - 61.7]	62.7 [59.1 - 66.5]	66.4 [62.7 - 70.3]*
60-69	32.4 [29.8 - 35.2]	35.1 [32.5 - 37.9]	39.5 [37.0 - 42.2]	37.5 [34.8 - 40.4]	37.4 [34.5 - 40.5]	44.2 [41.0 - 47.6]	45.6 [42.6 - 48.8]	45.3 [42.4 - 48.4]	54.0 [51.0 - 57.2]	55.2 [52.0 - 58.6]*
70-79	24.1 [21.3 - 27.3]	26.7 [23.8 - 30.0]	29.1 [26.2 - 32.3]	30.7 [27.8 - 33.9]	32.0 [29.2 - 35.1]	35.9 [32.8 - 39.3]	35.5 [32.0 - 39.4]	41.0 [37.5 - 44.8]	43.4 [39.9 - 47.2]	46.4 [43.0 - 50.1]*
80-89	21.8 [16.2 - 29.3]	25.9 [19.8 - 33.9]	22.2 [17.0 - 29.0]	24.9 [19.8 - 31.3]	26.8 [21.3 - 33.7]	32.9 [26.9 - 40.2]	29.0 [24.0 - 35.0]	30.2 [24.9 - 36.6]	38.0 [31.4 - 46.0]	30.4 [24.5 - 37.7]
**Age stand.**	**33.1 [31.5 - 34.8]**	**35.6 [33.9 - 37.3]**	**38.5 [36.9 - 40.1]**	**38.5 [36.9 - 40.1]**	**39.3 [37.7 - 41.0]**	**43.2 [41.5 - 45.1]**	**44.7 [43.0 - 46.5]**	**47.3 [45.5 - 49.1]**	**52.8 [50.9 - 54.7]**	**53.2 [51.4 - 55.1]***
